# Acceleration of Breathing

**Published:** 1878-05

**Authors:** 


ACCELERATION OF THE BREATHING is never absent in any case of actual consumption. In the last few weeks of life a few steps puts the patient out of breath, even if those steps be over a level floor. But long before this there was observed an inability to walk fast without considerable discomfort. In fact, a slow and measured tread is the symptom which first strikes the ordinary observer. The man himself may be scarcely an apparent invalid, except on close scrutiny. He may be lively in conversation, he may eat heartily, may have little or no cough, but any effort on your part to induce him to greater bodily activity is instinctively avoided. At a still earlier period one thing has been forced upon the attention of the patient: that he does not mount a pair of stairs with the same celerity as formerly. In days long ago he could take two or three steps at a stride, and even feel the better for it when he reached the top; but now, such an effort would make him puff and blow inconveniently. At an earlier period still, there is an observable feeling of tiredness about the legs and knees on going up stairs, a feeling of weakness there, not known in earlier years, implying a want of bodily vigor not pertinent to that stage of life.

It is to be hoped that no one will haste away with the impression that a little feeling of fatigue in going up a pair of stairs is a sign of consumption. This book is not written for quibbling critics; it is written for the instruction of people of sober views, who can look at a subject steadily, willing to be informed, but unwilling to run away with either end of the subject, or precipitate themselves into the weakness of extremes.

